# Microbial synthesis of a novel terpolyester P(LA‐*co*‐3HB‐*co*‐3HP) from low‐cost substrates

**DOI:** 10.1111/1751-7915.12453

**Published:** 2016-11-17

**Authors:** Yilin Ren, Dechuan Meng, Linping Wu, Jinchun Chen, Qiong Wu, Guo‐Qiang Chen

**Affiliations:** ^1^ Center for Synthetic and Systems BiologySchool of Life ScienceTsinghua‐Peking Center for Life SciencesTsinghua UniversityBeijing100084China; ^2^ Guangzhou Institutes of Biomedicine and HealthChinese Academy of SciencesGuangzhou510530People's Republic of China; ^3^ Center for Nano and Micro MechanicsTsinghua UniversityBeijing100084China; ^4^ MOE Key Lab of Industrial BiocatalysisDept Chemical EngineeringTsinghua UniversityBeijing100084China

## Abstract

Polylactide (PLA) is a bio‐based plastic commonly synthesized by chemical catalytic reaction using lactic acid (LA) as a substrate. Here, novel LA‐containing terpolyesters, namely, P[LA‐*co*‐3‐hydroxybutyrate (3HB)‐*co*‐3‐hydroxypropionate (3HP)], short as PLBP, were successfully synthesized for the first time by a recombinant *Escherichia coli* harbouring polyhydroxyalkanoate (PHA) synthase from *Pseudomonas stutzeri* (PhaC1_*Ps*_) with 4‐point mutations at E130D, S325T, S477G and Q481K, and 3‐hydroxypropionyl‐CoA (3HP‐CoA) synthesis pathway from glycerol, 3‐hydroxybutyryl‐CoA (3HB‐CoA) as well as lactyl‐CoA (LA‐CoA) pathways from glucose. Combining these pathways with the PHA synthase mutant phaC1_*Ps*_ (E130D S325T S477G Q481K), the random terpolyester P(LA‐*co*‐3HB‐*co*‐3HP), or PLBP, was structurally confirmed by nuclear magnetic resonance to consist of 2 mol% LA, 90 mol% 3HB, and 8 mol% 3HP respectively. Remarkably, the PLBP terpolyester was produced from low‐cost sustainable glycerol and glucose. Monomer ratios of PLBP could be regulated by ratios of glycerol to glucose. Other terpolyester thermal and mechanical properties can be manipulated by adjusting the monomer ratios. More PLBP applications are to be expected.

## Introduction

As increasing global warming and plastic pollution threaten human sustainability, materials from renewable biomass are attracting attention due to their biodegradability and environmentally friendliness. Polylactide (PLA) is a representative of bio‐based biodegradable polyester synthesized via combination of microbial lactic acid (LA) fermentation and chemical polymerization of lactide (Sudesh and Iwata, 2008; Nampoothiri *et al*., 2010; Park *et al*., 2012a,b). PLA has been used in areas of biomedical implants, food packaging and drug delivery. However, the complicated synthetic process including fermentation for LA production, LA purification, lactide esterification and lactide ring‐opening polymerization increases the cost, meanwhile the heavy metal residues in the final polymer products could limit its food or medical applications. In addition, PLA poor thermal and mechanical properties are also adverse to its large‐scale applications.

Polyhydroxyalkanoates (PHA) are a family of diverse polyesters synthesized by a variety of bacteria as intracellular carbon and energy storage compounds (Li *et al*., 2007; Chen and Hajnal, 2015; Koller and Rodríguez‐Contreras, 2015). As biorenewable and biodegradable materials, the diversity of PHA provides different physical properties to suit various applications (Chen, 2009; Brigham and Sinskey, 2012; Koller, 2014). Copolymerization of LA with other hydroxyalkanoate (HA) monomers via microbial synthesis is one of the effective methods to improve the physical properties of PLA or PHA (Li *et al*., 2010).

Recently, several engineered PHA synthases able to utilize lactyl‐CoA (LA‐CoA) and 3‐hydroxybutyric‐CoA (3HB‐CoA) as substrates were reported (Taguchi *et al*., 2008; Yang *et al*., 2011; Ochi *et al*., 2013). To deliver LA‐CoA in recombinant *Escherichia coli*, propionyl‐CoA transferases (Pct) in alanine fermentation pathway of several organisms including *Clostridium propionicum* and *Megasphaera elsdenii* were expressed in host strains respectively. A 6 mol% LA fraction in the copolyester was achieved.^15^ Subsequently, efforts were made on increasing the LA ratio in the copolyester by regulating the metabolic flux or evolving the PHA synthase (Jung *et al*., 2010; Yamada *et al*., 2010; Shozui *et al*., 2011), and also by the uses of different organisms in addition to commonly used *E. coli*, including *Corynebacterium glutamicum*,* Ralstonia eutropha* and *Sinorhizobium meliloti* (Song *et al*., 2012; Park *et al*., 2013; Tran and Charles, 2015). In addition, low‐cost carbon substrates, such as glucose and xylose, were applied to synthesize LA‐based copolyesters (Park *et al*., 2012a,b; Nduko *et al*., 2014; Salamanca‐Cardona *et al*., 2014), which could reduce production cost and facilitate its industrialization.

As mentioned, PLA has the major deficiencies of poor flexibility, ductility and thermal resistance, and copolymerization is possibly effective to improve the PLA properties. Therefore, LA copolyesters were investigated with P(LA‐*co*‐3HB) as a representative (Taguchi *et al*., 2008; Jung *et al*., 2010; Yamada *et al*., 2010; Shozui *et al*., 2011; Yang *et al*., 2011; Ochi *et al*., 2013). However, mechanical properties of PHB are similar to PLA, therefore, other monomers such as 3‐hydroxyvalerates (3HV), 3‐hydroxyhexanoate (3HHx) and glycolate (GA) were introduced into the LA copolyesters for property improvements (Shozui *et al*., 2010a,b; Choi *et al*., 2016; Li *et al*., 2016). Remarkably, poly(3‐hydroxypropionate) (P3HP), a relatively new PHA family member, has become very interesting due to its strong mechanical properties including an elongation at break of more than 600% and Young's modulus of 3 GPa (Andreeßen and Steinbüchel, 2010; Zhou *et al*., 2011a,b; Meng *et al*., 2012). Thus, 3HP monomers in LA copolyesters could very likely compensate for the shortcomings of PLA. This study attempted to biosynthesize a LA‐containing terpolyester consisting of LA, 3HP and 3HB with improved properties over PLA homopolyester.

## Results and discussion

### Engineering a pathway for biosynthesis of P(LA‐co‐3HB‐co‐3HP) from unrelated carbon source

**Figure 1:**
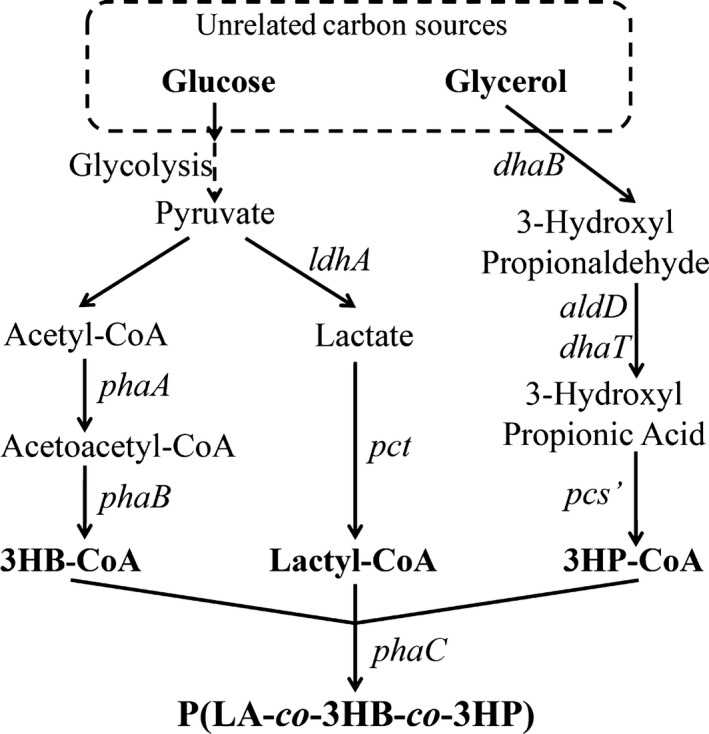
Metabolically engineered pathways for production of terpolyester P(LA‐*co*‐3HB‐*co*‐3HP) or PLBP from unrelated carbon source. Enzymes encoded by each gene are described below: *phaA*, β‐ketothiolase; *phaB*, NADPH‐dependent acetoacetyl‐CoA reductase; *ldhA*, lactate dehydrogenase; *pct*, propionyl‐CoA transferase; *dhaB*, glycerol dehydratase; *dhaT*, 1,3‐propanediol dehydrogenase; *aldD*, aldehyde dehydrogenase; *pcs*', propanoyl‐CoA synthetase; *phaC*, PHA synthase from *Pseudomonas stutzeri* strain 1317 (PhaC1_*Ps*_) with 4‐point mutations at E130D, S325T, S477G and Q481K.

For the above‐mentioned purpose, the substrate specificity of PHA synthase is the most important factor determining the monomer constituents incorporated into PHA. A mutant PHA synthase from *Pseudomonas stutzeri* strain 1317 (PhaC1_*Ps*_) with 4‐point mutations at E130D, S325T, S477G and Q481K was prepared as stated below, which could incorporate LA and other 3‐HAs into PLBP terpolyester. In addition, a 3HP synthetic pathway combined with LA and 3HB pathways were constructed to supply the monomers for the terpolyester synthesis (Fig. 1).

The engineering pathway allows adjustments of 3HB, LA and 3HP monomer ratios in the terpolyesters by feeding various ratios of glucose to glycerol. When a higher LA ratio is needed, LA can be added to the culture to supply additional LA precursor.

### Engineering a PHA synthase able to polymerize LA

**Figure 2:**
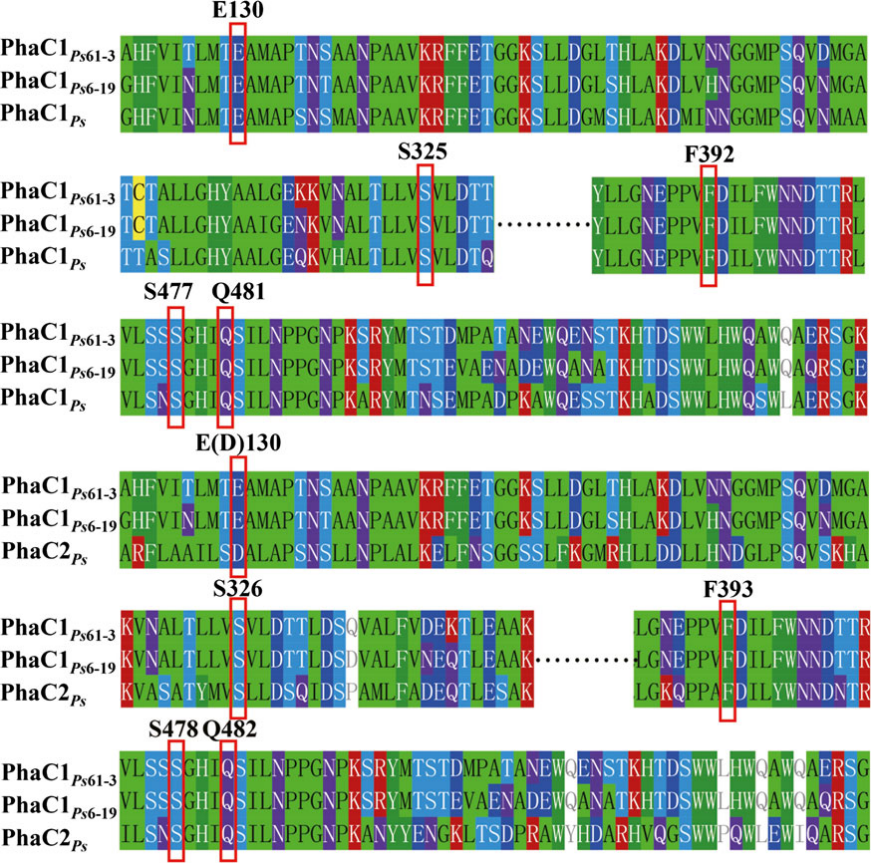
Alignment of *phaC1*_*Ps*_ and *phaC2*_*Ps*_ with previous LA polymerizing enzymes reported (Taguchi *et al*., 2008; Yamada *et al*., 2010; Yang *et al*., 2011). *phaC1*_*Ps*_ and *phaC2*_*Ps*_ from *Pseudomonas stutzeri* were aligned with reported LA polymerizing enzymes which were *phaC1*_*Ps61*‐3_ from *Pseudomonas sp*. 61‐3 and *phaC1*_*Ps6*‐19_ from *Pseudomonas sp*. MEBL6‐19. Several constitutive sites were found dominating the LA polymerizing capacity (in the red frames). Abbreviations: phaC1_*Ps*_, *Pseudomonas stutzeri* phaC1; phaC2_*Ps*_, *Pseudomonas stutzeri* phaC2; phaC1_*Ps*61‐3_, *Pseudomonas sp*. 61‐3 phaC1; phaC1_*Ps*6‐19_, *Pseudomonas sp*. MEBL6‐19 phaC1; E, glutamic acid; D, aspartic acid; S, serine; F, phenylalanine; Q, glutamine. Five mutation sites on *phaC1*_*ps*_ (*phaC2*_*ps*_), including E130D, S325(326)T, F392(393)S, S477(478)G and Q481(482)K, were selected based on the alignment result with the LA polymerizing enzymes.

The substrate specificity into PHA synthase is the most important factor determining the monomer constituents incorporated into PHA. Common PHA synthases are capable of polymerizing the 3‐hydroxyalkanoates (3HAs) CoA with variable carbon chain lengths (Bernd, 2003). LA, which is the monomer of PLA, is a 2HA, which is not accepted by natural PHA synthases. To synthesize PLBP terpolyesters, a mutant of PHA synthase, able to incorporate LA and other 3‐HAs at the same time was obtained via site‐directed mutagenesis on type II PHA synthase PhaC1_*Ps*_ of *P. stutzeri* 1317 which has already a versatile substrate specificity (Park *et al*., 2015). Five mutation sites on phaC1_*ps*_ (phaC2_*ps*_), including E130D, S325(326)T, F392(393)S, S477(478)G and Q481(482)K, were selected based on the alignment result with the previous LA‐polymerizing enzymes (Fig. 2) (Taguchi *et al*., 2008; Jung *et al*., 2010; Yamada *et al*., 2010; Yang *et al*., 2011; Chuah *et al*., 2013; Ochi *et al*., 2013). With various combinations of these site mutations, up to 20 *phaC1*_*Ps*_ and *phaC2*_*Ps*_ variants were constructed (Table S1). To compare their activities towards LA polymerization, the 3HB‐CoA synthetic pathway including genes of β‐ketothiolase (*phaA*), NADPH‐dependent acetoacetyl‐CoA reductase (*phaB*) of *R. eutropha*, LA‐CoA synthetic pathway including lactate dehydrogenase (*ldhA*) of *E. coli* and propionyl‐CoA transferase (*pct*) of *Clostridium propionicum* were constructed into a series of plasmids termed pBLPCAB‐X (X represents the specific *phaC* variant in the plasmid). pBLPCAB‐Xs were derived from pBHR68 (kindly donated by Prof Steinbűchel of Műnster Univ/Germany), with the three genes of *ldhA*,* pct* and *phaC* variant inserted downstream the P_*re*_ promoter. The LA polymerization activities of typical *phaC* variants were determined by expressing the corresponding plasmids in *E. coli* S17‐1 (Table 1).

**Table 1:** LA polymerization activities of typical *phaC* variants

*E. coli*	*phaC*_*Ps*_ variants	CDM (g L^−1^)	PHA/CDM (wt%)	LA (mol%)
S‐pBLPCAB1	*phaC1*_*Ps*_	4.03 ± 0.27	39.22 ± 0.58	0
S‐pBLPCAB1‐2	*phaC1*_*Ps*_ (Q481K S325T)	4.18 ± 0.13	40.12 ± 0.23	2.81 ± 0.30
S‐pBLPCAB1‐4	*phaC1*_*Ps*_ (Q481K S325T E130D S477G)	3.13 ± 0.31	36.94 ± 0.38	5.01 ± 1.24
S‐pBLPCAB1‐5	*phaC1*_*Ps*_ (Q481K S325T E130D S477G F392S)	3.01 ± 0.22	37.51 ± 0.23	3.01 ± 0.72
S‐pBLPCAB2	*phaC2*_*Ps*_	3.24 ± 0.09	41.50 ± 0.61	0
S‐pBLPCAB2‐2	*phaC2*_*Ps*_ (Q482K S326T)	4.15 ± 0.43	37.71 ± 0.49	0
S‐pBLPCAB2‐3	*phaC2*_*Ps*_ (Q482K S326T S478G)	3.76 ± 0.52	36.13 ± 0.77	0

Recombinant strains were cultivated for 48 h in shake flasks. The data are the averages of three parallel experiments.

LA, lactate; CDM, cell dry mass.

When analysing the PHA accumulation capacity, single‐point mutation on *phaC*_*Ps*_ was found insufficient for LA polymerizations (data not shown). By combining Q481K and S325T mutations in *phaC1*_*Ps*_, the recombinant started to synthesize P(2.81% LA‐*co*‐3HB) copolyester, confirming two‐point mutant *phaC1*_*Ps*_ (Q481K S325T) capable of polymerizing LA into PHA. Additional mutations on E130D and S477G to the above two points mutant increased the LA specificity as the LA ratio in the PHA copolyester produced by the recombinant expressing *phaC1*_*Ps*_ (Q481K S325T E130D S477G) increased to over 5% (Table 1). However, mutation F392S had a negative effect on LA incorporation, as F392S added to *phaC1*_*Ps*_ (Q481K S325T E130D S477G) reduced LA ratio in the copolyester to 3% (Table 1). Interestingly, *phaC2*_*Ps*_ showed no activity towards LA‐CoA no matter what point mutations were introduced. As a result, the LA polymerizing mutant enzyme PhaC1_*Ps*_ (Q481K S325T E130D S477G) with the highest efficiency was obtained compared with other *phaC* wild type or mutant enzymes (Table 1 and Table S2). We therefore named the plasmid pBLPCAB1‐4‐containing *phaC1*_*Ps*_ (Q481K S325T E130D S477G) plasmid pLA in further studies.

### Construction of an effective PLBP synthetic system

Aimed to produce a novel terpolyester PLBP, three synthetic pathways for each constituent were constructed (Fig. 1), including PHB and PLA synthetic routes that were reported. The P3HP synthetic pathway was focused on as it would improve the mechanical properties of the terpolyesters.

P3HP could be synthesized from 1, 3‐propanediol, glycerol and glucose (Andreeßen and Steinbüchel, 2010; Zhou *et al*., 2011a,b; Meng *et al*., 2015). Glycerol was chosen in this study to regulate 3HP ratio in the copolyesters, whereas glucose was the substrate for PLA and 3HB synthesis. The 3HP synthetic pathway from glycerol consisted of genes encoding glycerol dehydratase (*dhaB*) of *Klebsiella pneumoniae*, 1,3‐propanediol dehydrogenase (*dhaT*) and aldehyde dehydrogenase (*aldD*) of *Pseudomonas putida* KT2442 and ACS domain of tri‐functional propionyl‐CoA synthetase (*pcs*') functioning as a CoA ligase of *Chloroflexus aurantiacus* (Andreeßen and Steinbüchel, 2010; Zhou *et al*., 2011a,b; Meng *et al*., 2012). The p3HP1p plasmid was constructed based on the pSEVA351 for P3HP production and contained genes of *dhaT*,* aldD*,* dhaB* and *pcs*' (Silva‐Rocha *et al*., 2013). Gene fragments *dhaT‐aldD*,* pcs* and *dhaB* were amplified from plasmid pZQ03, pDC02 and pZQ01 respectively. Subsequently, the pSEVA351 backbone was ligated with these three fragments by Gibson assembly. When strengthen the expression of dominant gene *dhaB* controlling glycerol utilization, the optimized plasmid p3HP2p exhibited enhanced efficiency in P3HP synthesis when it was co‐expressed with pBHR68 in recombinant *E. coli* S17‐1 (Table S3).

Finally, recombinant *E. coli* S‐LA‐3HP harbouring plasmids pLA and p3HP2p was obtained. When cultivated in LB medium supplemented with 20 g L^−1^ glucose and 10 g L^−1^ glycerol, a new terpolyester P(90.41 mol% 3HB‐*co*‐7.78 mol% 3HP‐*co*‐1.81 mol% LA) was successfully synthesized for the first time from unrelated carbon sources glucose and glycerol.

### Nuclear magnetic resonance analyses of the PLBP terpolyester

**Figure 3:**
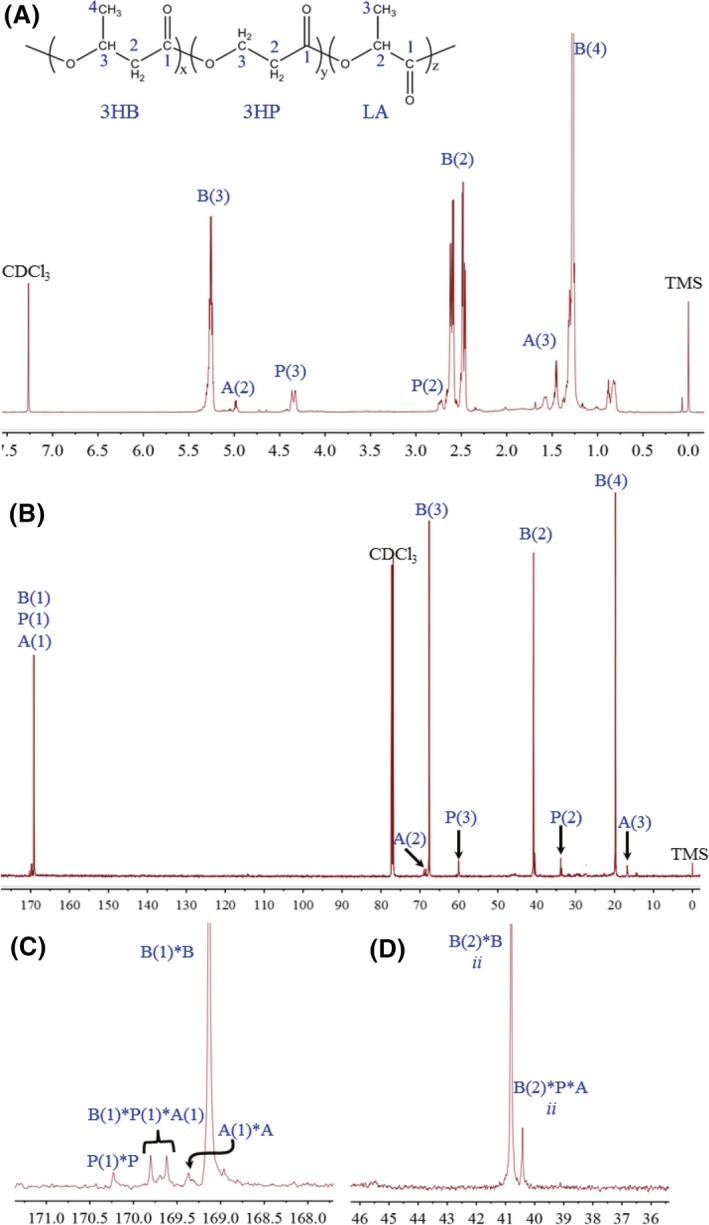
NMR analysis of PLBP terpolyester. ^1^H NMR spectra (A) and ^13^C NMR spectra (B) of random copolyester P(3HB‐*co*‐3HP‐*co*‐LA) containing 90.41 mol% 3HB, 7.78 mol% 3HP and 1.81 mol% LA, respectively, and its expanded ^13^C NMR spectra of carboxyl carbon [B(1), P(1), A(1)] area (C) and methylene regions (D) in the terpolyester. B, P and A refer to 3HB, 3HP and LA; numbering schemes were the same as molecular formulations of polyester indicated in the inset in (A). N*M represents the interaction of monomer N and M. “i” indicates “isotactic.” Chemical shifts are in ppm and tetramethylsilane (TMS) was employed as an internal chemical shift standard.

The composition and monomer sequence distribution of P(3HB‐*co*‐3HP‐*co*‐LA) was confirmed by nuclear magnetic resonance (NMR) (Fig. 3). From the ^1^H NMR spectra (Fig. 3A), there were not only well‐characterized proton resonances of B(2), B(3) and B(4) in 3HB units (3HB abbreviated as B, 3HP monomer abbreviated as P and LA abbreviated as A) but also additional four proton resonances assigned to the 3HP units such as P(2), P(3) and LA units including A(2) and A(3) with identical intensities based on the previous studies (Park *et al*., 2013, 2015; Meng *et al*., 2015). The molar ratio of 3HB, 3HP and LA in the PHA copolyester was 90.41%, 7.78% and 1.81%, respectively, as calculated using the intensity of B(3), P(3) and A(2). The individual carbon species in 3HB, 3HP and LA monomer were also identified by specific ^13^C NMR (Fig. 3B). The expanded spectra of individual splitting resonance of carboxyl carbon B(1), P(1) and A(1) in the copolyester were split into multiple peaks (Fig. 3C), which were assigned to the 3HB‐centred (B(1)*B), 3HP‐centred (P(1)*P), LA‐centred (A(1)*A) and the three units comonomer sequences [B(1)*P(1)*A(1)] (N*M represents the interaction of monomer N and M) (Meng *et al*., 2015; Park *et al*., 2015). This phenomenon was due to different sequence arrangements of 3HB, 3HP and LA monomers in the polymer chains, which is common in random PHA copolyesters (Hu *et al*., 2011; Tripathi *et al*., 2012).

The tacticity distribution of tri‐block copolyester was studied via ^13^C NMR based on various stereo‐sequences. The good resolution of methylene regions B(2) was chosen as an example for analysis. Two sharp peaks corresponding to B(2)*B and B(2)*P*A were observed (Fig. 3D). No split in each peak was visible, indicating that the stereo‐sequence was isotactic. If the polymer is syndiotactic or atactic, some diad or quadruple peaks should be observed in ^13^C NMR (Kemnitzer *et al*., 1993; Hocking and Marchessault, 1995). The above detailed analysis of ^1^H NMR and ^13^C NMR spectra clearly indicated that PHA was a random copolyester P(3HB‐*co*‐3HP‐*co*‐LA) with an isotactic microstructure.

### Regulation of the monomer ratios in PLBP terpolyesters

Recombinant *E. coli* S17‐1 harbouring genes described in Fig. 1 could produce terpolyesters PLBP consisting of LA, 3HB and 3HP when grown in a mixture of glucose and glycerol as substrates (Table 2). The terpolyesters PLBP were fully synthesized from unrelated carbon sources, which was more economic for PHA synthesis (Hermann‐Krauss *et al*., 2013; Povolo *et al*., 2013). Compositions of monomers LA, 3HB and 3HP in PLBP could be adjusted by changing the glucose‐to‐glycerol ratios.

**Table 2:** PLBP production using various substrate concentrations by *E. coli* S17‐1 *ΔpflA* harbouring two plasmids of pLA' and p3HP2p

Glu (g L^−1^)	Gly (g L^−1^)	LA (g L^−1^)	CDM (g L^−1^)	PHA (wt%)	3HP (mol%)	LA (mol%)
20	10	0	3.70 ± 0.12	40.94 ± 0.44	9.85 ± 1.43	10.74 ± 2.22
20	5	0	3.88 ± 0.3	45.17 ± 1.08	8.04 ± 0.77	13.10 ± 1.23
20	2	0	4.79 ± 0.08	53.31 ± 2.12	5.42 ± 0.8	9.18 ± 0.31
30	10	0	5.21 ± 0.72	59.04 ± 1.07	8.93 ± 0.87	11.53 ± 2.15
10	10	0	3.05 ± 0.41	38.67 ± 1.7	12.03 ± 0.39	0.79 ± 0.55
20	10	2	1.43 ± 0.19	45.06 ± 3.50	10.27 ± 0.69	23.54 ± 1.95
20	10	5	0.98 ± 0.23	42.67 ± 0.56	8.95 ± 2.3	27.78 ± 0.72

The recombinant was cultivated in LB medium supplemented with different concentrations of glucose (glu), glycerol (gly) and LA for 48 h in shake flasks. The data are the averages of three parallel experiments.

CDM, cell dry mass; PHA, the terpolyester; 3HP, 3‐hydroxypropionate; LA, lactate.

Lactic acid ratio was as low as 1.81% in the terpolyester when PLBP was produced by the wild‐type *E. coli* S17‐1 harbouring genes described in Fig. 1. To increase the LA ratio in PLBP, the synthetic pathway was optimized and the competitive pathways of LA were deleted. Gene *ldhA* was inserted in pLA plasmid and was overexpressed to produce more LA as *ldhA* functions in converting pyruvate into LA (Jung *et al*., 2010). However, it was found that overexpression of *ldhA* alone could not enhance the LA content in the terpolyester (Table S4) as a gene *dld* could convert LA back into pyruvate (Dym *et al*., 2000; Choi *et al*., 2016). Unexpectedly, the recombinant strain‐containing plasmid pLA' without *ldhA*, replacing pLA plasmid of the PLBP synthetic system, synthesized a PLBP terpolyester with an increased LA ratio under the same culture conditions (Fig. S1.2). The deletion of *ldhA* in pLA plasmid resulted in a transcriptional change in those genes downstream the same promoter for the ones that were closer to the promoter (Fig. S1.1). It was proven that the transcriptional level of *pct* was correlated with the LA ratio in PLBP terpolyesters from the RT‐PCR results (Fig. S1.2). It suggested that PCT‐mediated CoA transfer reaction that transformed LA into LA‐CoA was the time‐limiting step of LA polymerization. Meanwhile, to improve LA production, a competitive pathway, pyruvate–formate pathway, was weakened. Gene *pflA*, the activator of pyruvate–formate lyase, was knocked out in *E. coli* S17‐1 (Zhu and Shimizu, 2004; Shozui *et al*., 2010b; Zhou *et al*., 2011a,b). The gene *pflA* knockout lead to an obvious decrease in formate production of the strain, whereas the lactate concentration in the culture medium increased oppositely (Fig. S2).

**Figure 4:**
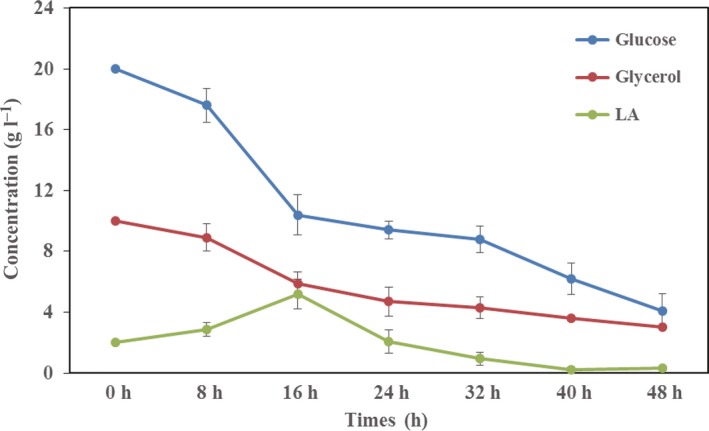
The concentration of glucose, glycerol and lactate in shake‐flask studies. *E. coli* S17‐1 Δ*pflA* harbouring two plasmids of pLA' and p3HP2p was cultured in LB medium supplemented with 20 g L^−1^ glucose, 10 g L^−1^ glycerol and 2 g L^−1^ lactate. Blue line, glucose; red line, glycerol; green line, lactate. Error bars represent the standard deviation of experiments conducted in triplicates.

*Escherichia coli* S17‐1 Δ*pflA* harbouring two plasmids of pLA' and p3HP2p was constructed and studied in shake flasks using different substrates combinations (Table 2 and Fig. 4). Obviously, the LA ratio in PLBP terpolyester produced by this recombinant was increased compared with the wild‐type *E. coli* S17‐1. The substrates added to the culture were utilized simultaneously indicating the synthesis of random copolyesters (Fig. 4). In general, several tendencies could be summarized from the data: there was a positive correlation between LA ratio and glucose concentration. Especially when glucose concentration was decreased to 10 g L^−1^, the LA ratio was down to 0.79% because most of the glucose was utilized for cell growth; when extra D, L‐LA was added into the medium, LA ratio increased sharply to more than 27%, whereas the cell dry mass (CDM) was deceased oppositely due to the toxicity of LA; as the substrate of P3HP, glycerol affected 3HP ratio in PLBP terpolyester in a similar relationship as that between glucose and LA ratio. Interestingly, 3HP ratio reached its peak value of 12.03% when glucose was reduced to 10 g L^−1^. Synthesis of PLBP terpolyester with variable compositions was achieved.

At a high glucose concentration of 30 g L^−1^, cells grew to over 5.2 g L^−1^ containing close to 60% PHA, this was both the highest cell growth and the highest PHA accumulation compared with other low glucose concentration (Table 2). The terpolyester consisted of 9% 3HP, 12% LA and 79% 3HB. The result indicated that high concentration of glucose favoured formation of cell mass and PHA, especially PHB. At the lowest glycerol concentration of 2 g L^−1^, 3HP had the lowest content of 5% in the terpolyester. LA addition improved LA ratios in the terpolyester, but it had negative effect on cell growth (Table 2). Hence, several strategies exist to change the composition of the monomer level by adjusting the ratio of fed carbon sources.

### Physical characterization of PLBP with different monomer compositions

Two types of PLBPs were extracted and the compositions of them were determined by GC. The physical properties of PLBPs including molecular mass, thermal properties and mechanical properties were studied (Table 3). The weight average molecular mass of PLBP was less than 2 × 10^5^, which was lower compared with its homopolyester and P(LA‐*co*‐3HB) copolyester, yet approximated the same as weight average molecular mass of PLBV terpolyesters (Shozui *et al*., 2010b). In addition, PLBPs inherited the ductility of P3HP as it had an improved elongation at break of over 100% when the 3HP ratio reached 15%. The thermal parameters including *T*_*m*_, *T*_*g*_ and Δ*H*_*m*_ of PLBP were in between of those of individual homopolyesters (PLA, P3HB and P3HP) as blocks. Interestingly, PLBPs had two *T*_*m*_ and the lower one was close to the *T*_*m*_ of PHB. Possibly, the presence of 3HB‐rich segments in the terpolyester led to this phenomenon. The incorporation of 3HP into P(LA‐*co*‐3HB) significantly improved the tensile strength and elongation at break compared with its copolyester P(LA‐*co*‐3HB).

**Table 3:** Physical characterization of various PLBP terpolyesters

Monomer ratio^a^ (mol%)			Molecular mass^b^			Mechanical properties^c^			Thermal properties^d^		
LA	3HB	3HP	*M*_*w*_ (10^4^)	*M*_*n*_ (10^4^)	*M*_*w*_ */M*_*n*_	Tensile strength (MPa)	Young's modulus (MPa)	Elongation at break (%)	*T*_*g*_ (°C)	*T*_*m*_ (°C)	Δ*H*_*m*_ (J g^−1^)
100^e^	0	0	20	–	–	52 ± 2	1020	2	60	153	9.2
0	100^f^	0	–	–	–	18 ± 0.7	1470 ± 78	3 ± 0.4	7.1	131.8	–
0	0	100^g^	–	30	–	28.3	333.3	683.5	−21.5	78	54
15^h^	85	0	82	34.2	2.4	10 ± 0	194 ± 5	75 ± 2	−9, 19	149, 167	0.6, 3.2
1.8	90.4	7.8^i^	15.3	9.1	1.68	18 ± 2	332.8 ± 8.5	15.3 ± 4.5	−5.9	129, 150	3.3, 32
7.2	79.8	13^j^	11.7	7.6	1.54	12.5 ± 1.3	231.4 ± 9.7	100.9 ± 12	−2	132, 154	2.7, 39

a Determined by gas chromatography.

b *M*_*w*_, weight‐averaged Molecular mass; *M*_*n*_, number‐averaged Molecular mass; *M*_*w*_ */M*_*n*_; polydispersity; the unit of *M*_*w*_ and *M*_*n*_ is Da.

c The values are the averages of at least three independent measurements.

d *T*_*g*_, glass‐transition temperature; *T*_*m*_, melting temperature; Δ*H*_*m*_, enthalpy of fusion.

e PLA was chemically synthesized (Zaman *et al*., 2011).

f P3HB was synthesized by bacteria (Li *et al*., 2011).

g P3HP was synthesized by bacteria (Zhou *et al*., 2011a,b).

h P(LA‐*co*‐3HB) was produced by recombinant *E. coli* (Yamada *et al*., 2011).

i Sample weight of P(90.4 mol% 3HB‐co‐7.8 mol% 3HP‐co‐1.8 mol% LA) was 18.2 mg.

j Sample weight of P(79.8 mol% 3HB‐co‐13 mol% 3HP‐co‐7.2 mol% LA) was 17.5 mg.

## Conclusions

Recombinant *E. coli* S17‐1 Δ*pflA* consisting of three synthetic pathways of lactyl‐CoA, 3‐hydroxypropionyl‐CoA and 3‐hydroxybutyryl‐CoA was able to polymerize the three monomers to form random terpolyester P(LA‐*co*‐3HB‐*co*‐3HP) or PLBP under the catalysis of *phaC1*_*Ps*_ (Q481K S325T E130D S477G) cloned from *P. stutzeri* strain 1317 with four specific point mutations. The terpolyester compositions can be controlled by changing the ratios of three substrates including glucose, glycerol and LA. The terpolyesters have changing properties depending on monomer ratios, especially an enhanced elongation at break compared with the homopolyesters of 3HB and LA as well as their copolyesters.

## Experimental procedures

### Microorganism, plasmid and shake‐flask culture conditions

All the microorganisms and plasmids used in this study are listed in Table 4. *E. coli* Trans 1T1 and JM109 were used as the host strains for genetic manipulation. *E. coli* S17‐1 was used for polymer production (Simon *et al*., 1983). All the *E. coli* strains were cultured in LB medium. LB medium contains (g L^−1^): 10 tryptone, 5 yeast extract and 10 NaCl. Glycerol and glucose were added into LB medium as carbon sources. The seed cultures stored at −80°C were inoculated into LB medium and cultivated at 37°C for 12 h at 200 rpm/min on a rotary shaker for reactivation (BBT‐14‐BJQH042, INFORS HT, Hong Kong, China). Subsequently, the seed cultures were inoculated into 500 mL conical flasks containing 50 mL LB medium with an inoculation volume ratio of 5%. When an antibiotic selection pressure was required, the medium was supplemented with ampicillin (100 μg mL^−1^), kanamycin (50 μg mL^−1^) or chloramphenicol (34 μg mL^−1^). To increase the LA ratio in the terpolyester, various amounts of D, L‐LA (1, 2 or 5 g L^−1^) were added into the LB medium along with NaOH‐modulating pH to neutral. When P3HP was the constituent of the polymer, 5 μM vitaminB_12_ (VB_12_) was added into the medium to maintain the activity of glycerol dehydratase (*dhaB*).

**Table 4:** Strains and plasmids used in this study

Strains/plasmids	Description	Reference/source
*E. coli* Trans1‐T1	Expression host	TransGen Biotech
*E. coli* S17‐1	*recA*, harbours the *tra* genes of plasmid RP4 in the chromosome; *proA*,* thi‐1*	Simon *et al*. (1983)
S‐NC	*E. coli* S17‐1 harbours pBluescript SK^−^ plasmid	This study
S‐BL	*E. coli* S17‐1 harbours pBL plasmid	This study
S‐LA	*E. coli* S17‐1 harbours pLA plasmid	This study
S‐LA'	*E. coli* S17‐1 harbours pLA' plasmid	This study
pBHR68	Derivative of pBluescript SK^−^ containing the 5.2‐kb *SmaI/EcoRI* fragment comprising the PHA operon from *Ralstonia eutropha*	Spiekermann *et al*. (1999)
pSEVA351	Cloning vector, RSF1010 replicon, Cm^R^	Silva‐Rocha *et al*. (2013)
pBluescript SK^−^	The commonly used commercial plasmid	TransGenBiotech
pZQ 03	Derivative of pBHR68, *phaC* and *pcs*' under the control of *lac* promoter, Amp^R^	Zhou *et al*. (2011a,b)
pDC02	Derivative of pBHR68, *phaC*,* dhaB*,* gpp*,* gpd*,* gdrAB* and *pduP* under the control of P_*Re*_ promoter derived from *Ralstonia eutropha pha* operon, Amp^R^	Meng *et al*. (2015)
pLA	Derivative of pBHR68, *phaC1*_*Ps*_ (S325T Q481K E130D S477G), *pct* and *ldhA* was inserted into backbone	This study
pLA'	Derivative of pBHR68, *phaC1*_*Ps*_ ((S325T Q481K E130D S477G) and *pct* was inserted into backbone	This study
p3HP1p	Derivative of pSEVA351, *dhaB*,* dhaT*,* aldD* and *pcs*' was inserted into backbone downstream 1 P_*re*_ promoters	This study
p3HP2p	Derivative of pSEVA351, *dhaB*,* dhaT*,* aldD* and *pcs*' was inserted into backbone with 2 P_*re*_ promoters	This study
pBL	Derivative of pBluescript SK^−^, *ldhA* was inserted into backbone	This study

### PHA analysis, extraction and purification

Cells were harvested by centrifugation (CR21 GIII; Hitachi, Tokyo, Japan) at 10 000 rpm/min for 8 min, then washed with distilled water and centrifuged again. CDM were measured after the concentrated cells were lyophilized at −65°C with five times air pressure (LGJ‐10C; SiHuanKeXue, Beijing, China). Thirty to fourty milligrams of lyophilized cells was used for the transesterification reaction in which 2 mL of transesterification mixture and 2 mL of chloroform were added in each transesterification test tube (Kato *et al*., 1996). After a 4 h transesterification at 100°C, the PHA content and monomer compositions of the cells were assayed by gas chromatograph (GC‐2014; Shimadzu, Suzhou, China) (Ouyang *et al*., 2007). The intracellular polymers were extracted using a Soxhlet extractor (Soxtec 2050; Foss, Hilleroed, Denmark). The extracted PHA was purified via precipitation when mixed with the 10‐folds volume of ice‐cold ethanol and dissolved in chloroform for film casting.

### Metabolic flux analysis

Concentrations of LA, glycerol and glucose were determined using a high‐performance liquid chromatography (HPLC) (LC‐20A; Shimadzu) equipped with an ion exchange column (Aminexs HPX‐87H; Bio‐Rad, 7.8 × 300 mm^2^, Hercules, California, USA) and a refractive index detector (RID‐10A; Shimadzu). Gene *pct* transcriptional level was assayed using RT‐PCR. Total RNA was extracted from recombinant *E. coli* strains using RNA prep pure Cell/Bacteria Kit (Tiangen, Beijing, China). cDNA was synthesized using Fastquant RT Kit (Tiangen) and then real‐time PCR (RT‐PCR) was carried out for mRNA analysis with SuperReal PreMix (SYBR Green; Tiangen). 16S rRNA was used as the inner standard. The experimental procedures were described (Lv *et al*., 2015).

### NMR analysis on PHA

The ^1^H and ^13^C spectra were obtained using a JEOL JNM‐ECA 600 NMR spectrometer to measure the polymer compositions, the chemical microstructures and the monomer sequences. Tetramethylsilane was used as the internal standard.

### Molecular mass and other properties of PHA

Molecular mass was determined via gel permeation chromatography (GPC Spectra System P2000; Shimadzu) equipped with a Shimadzu HSG60 column at 40°C. The melting temperature (*T*_*m*_), enthalpy of fusion (Δ*H*_*m*_) and glass‐transition temperature (*T*_*g*_) were measured via differential scanning calorimetry (DSC‐60; Shimadzu) in a temperature ranging from −80°C to 200°C under a nitrogen atmosphere of 50 mL min^−1^. Thermal stabilities of the materials were studied by a thermogravimetric analyser (TA‐Q50; TA Instrument, New Castle, Delaware, USA). Three to five milligrams of each sample was loaded at temperature ranging from 10 to 400°C in a nitrogen atmosphere of 50 mL min^−1^ (Shen *et al*., 2009). Mechanical properties were studied using a materials testing machine (INSTRON 3365; Instron, Grove City, Ohio, USA) at room temperature at a speed of 5 mm min^−1^.

## Supporting information


**Table S1.** Site specific mutations of two PHA synthases *phaC*_*Ps*_ variants.
**Table S2.** Comparison of various LA polymerizing enzymes.
**Table S3.** P3HP synthetic ability of *E. coli* harboring p3HP1p and p3HP2p plasmids, respectively.
**Table S4.** Effects of expressing gene *ldhA* on LA synthesis.
**Fig. S1.1.** Comparison of plasmids structure between pLA and pLA'.
**Fig. S1.2.** Relationship between *pct* transcriptional level and LA ratio in the terpolymer.
**Fig. S2.** Formations of extracellular formate and lactate by *E. coli* S17‐1 (A) and the Δ*pflA* mutant (B) under aerobic conditions.

## References

[mbt212453-bib-0001] Andreeßen, B. , and Steinbüchel, A. (2010) Biosynthesis and biodegradation of 3‐hydroxypropionate‐containing polyesters. Appl Environ Microbiol 76: 4919–4925. 20543057 10.1128/AEM.01015-10 PMC2916506

[mbt212453-bib-0002] Bernd, H. (2003) Polyester synthases: natural catalysts for plastics. Biochem J 376: 15–33. 12954080 10.1042/BJ20031254 PMC1223765

[mbt212453-bib-0003] Brigham, C.J. , and Sinskey, A.J. (2012) Applications of polyhydroxyalkanoates in the medical industry. Int J Biotechnol Wellness Ind 1: 52.

[mbt212453-bib-0004] Chen, G.Q. (2009) A microbial polyhydroxyalkanoates (PHA) based bio‐ and materials industry. Chem Soc Rev 38: 2434–2446. 19623359 10.1039/b812677c

[mbt212453-bib-0005] Chen, G.‐Q. , and Hajnal, I. (2015) The ‘PHAome’. Trend Biotechnol 33: 559–564. 10.1016/j.tibtech.2015.07.006 26409775

[mbt212453-bib-0006] Choi, S.Y. , Park, S.J. , Kim, W.J. , Yang, J.E. , Lee, H. , Shin, J. , and Lee, S.Y. (2016) One‐step fermentative production of poly (lactate‐co‐glycolate) from carbohydrates in *Escherichia coli* . Nat Biotechnol 34: 435–440. 26950748 10.1038/nbt.3485

[mbt212453-bib-0007] Chuah, J.‐A. , Tomizawa, S. , Yamada, M. , Tsuge, T. , Doi, Y. , Sudesh, K. , and Numata, K. (2013) Characterization of site‐specific mutations in a short‐chain‐length/medium‐chain‐length polyhydroxyalkanoate synthase: in vivo and in vitro studies of enzymatic activity and substrate specificity. Appl Environ Microb 79: 3813–3821. 10.1128/AEM.00564-13 PMC3675958 23584780

[mbt212453-bib-0008] Dym, O. , Pratt, E.A. , Ho, C. , and Eisenberg, D. (2000) The crystal structure of D‐lactate dehydrogenase, a peripheral membrane respiratory enzyme. Proc Natl Acad Sci USA 97: 9413–9418. 10944213 10.1073/pnas.97.17.9413 PMC16878

[mbt212453-bib-0009] Hermann‐Krauss, C. , Koller, M. , Muhr, A. , Fasl, H. , Stelzer, F. , and Braunegg, G. (2013) Archaeal production of polyhydroxyalkanoate (PHA) co‐ and terpolyesters from biodiesel industry‐derived by‐products. Archaea 2013: 465–466. 10.1155/2013/129268 PMC3880725 24453697

[mbt212453-bib-0010] Hocking, P.J. , and Marchessault, R.H. (1995) Microstructure of Poly [(R, S)‐. beta.‐hydroxybutyrate] by 13C NMR. Macromolecules 28: 6401–6409.

[mbt212453-bib-0011] Hu, D. , Chung, A.‐L. , Wu, L.‐P. , Zhang, X. , Wu, Q. , Chen, J.‐C. , and Chen, G.‐Q. (2011) Biosynthesis and characterization of polyhydroxyalkanoate block copolymer P3HB‐b‐P4HB. Biomacromolecules 12: 3166–3173. 21863836 10.1021/bm200660k

[mbt212453-bib-0012] Jung, Y.K. , Kim, T.Y. , Park, S.J. , and Lee, S.Y. (2010) Metabolic engineering of *Escherichia coli* for the production of polylactic acid and its copolymers. Biotechnol Bioeng 105: 161–171. 19937727 10.1002/bit.22548

[mbt212453-bib-0013] Kato, M. , Bao, H. , Kang, C.‐K. , Fukui, T. , and Doi, Y. (1996) Production of a novel copolyester of 3‐hydroxybutyric acid and medium‐chain‐length 3‐hydroxyalkanoic acids by *Pseudomonas* sp. 61‐3 from sugars. Appl Microbiol Biotechnol 45: 363–370.

[mbt212453-bib-0014] Kemnitzer, J.E. , McCarthy, S.P. , and Gross, R.A. (1993) Preparation of predominantly syndiotactic poly (. beta.‐hydroxybutyrate) by the tributyltin methoxide catalyzed ring‐opening polymerization of racemic. beta.‐butyrolactone. Macromolecules 26: 1221–1229.

[mbt212453-bib-0015] Koller, M. (2014) Poly (hydroxyalkanoates) for food packaging: application and attempts towards implementation. Appl Food Biotechnol 1: 3–15.

[mbt212453-bib-0016] Koller, M. , and Rodríguez‐Contreras, A. (2015) Techniques for tracing PHA‐producing organisms and for qualitative and quantitative analysis of intra‐and extracellular PHA. Eng Life Sci 15: 558–581.

[mbt212453-bib-0017] Li, R. , Zhang, H. , and Qi, Q. (2007) The production of polyhydroxyalkanoates in recombinant *Escherichia coli* . Bioresour Technol 98: 2313–2320. 17097289 10.1016/j.biortech.2006.09.014

[mbt212453-bib-0018] Li, Z.‐J. , Shi, Z.‐Y. , Jian, J. , Guo, Y.‐Y. , Wu, Q. , and Chen, G.‐Q. (2010) Production of poly (3‐hydroxybutyrate‐co‐4‐hydroxybutyrate) from unrelated carbon sources by metabolically engineered *Escherichia coli* . Metab Eng 12: 352–359. 20304089 10.1016/j.ymben.2010.03.003

[mbt212453-bib-0019] Li, S.Y. , Dong, C.L. , Wang, S.Y. , Ye, H.M. , and Chen, G.‐Q. (2011) Microbial production of polyhydroxyalkanoate block copolymer by recombinant *Pseudomonas putida* . Appl Microbiol Biotechnol 90: 659–669. 21181145 10.1007/s00253-010-3069-2

[mbt212453-bib-0020] Li, Z.‐J. , Qiao, K. , Shi, W. , Pereira, B. , Zhang, H. , Olsen, B.D. , and Stephanopoulos, G. (2016) Biosynthesis of poly (glycolate‐co‐lactate‐co‐3‐hydroxybutyrate) from glucose by metabolically engineered *Escherichia coli* . Metab Eng 35: 1–8. 26778413 10.1016/j.ymben.2016.01.004

[mbt212453-bib-0021] Lv, L. , Ren, Y.‐L. , Chen, J.‐C. , Wu, Q. , and Chen, G.‐Q. (2015) Application of CRISPRi for prokaryotic metabolic engineering involving multiple genes, a case study: controllable P (3HB‐co‐4HB) biosynthesis. Metab Eng 29: 160–168. 25838211 10.1016/j.ymben.2015.03.013

[mbt212453-bib-0022] Meng, D.‐C. , Shi, Z.‐Y. , Wu, L.‐P. , Zhou, Q. , Wu, Q. , Chen, J.‐C. , and Chen, G.‐Q. (2012) Production and characterization of poly (3‐hydroxypropionate‐co‐4‐hydroxybutyrate) with fully controllable structures by recombinant *Escherichia coli* containing an engineered pathway. Metab Eng 14: 317–324. 22561235 10.1016/j.ymben.2012.04.003

[mbt212453-bib-0023] Meng, D.‐C. , Wang, Y. , Wu, L.‐P. , Shen, R. , Chen, J.‐C. , Wu, Q. , and Chen, G.‐Q. (2015) Production of poly (3‐hydroxypropionate) and poly (3‐hydroxybutyrate‐co‐3‐hydroxypropionate) from glucose by engineering *Escherichia coli* . Metab Eng 29: 189–195. 25842374 10.1016/j.ymben.2015.03.015

[mbt212453-bib-0024] Nampoothiri, K.M. , Nair, N.R. , and John, R.P. (2010) An overview of the recent developments in polylactide (PLA) research. Bioresour Technol 101: 8493–8501. 20630747 10.1016/j.biortech.2010.05.092

[mbt212453-bib-0025] Nduko, J.M. , Matsumoto, K.I. , Ooi, T. , and Taguchi, S. (2014) Enhanced production of poly (lactate‐co‐3‐hydroxybutyrate) from xylose in engineered *Escherichia coli* overexpressing a galactitol transporter. Appl Microbiol Biotechnol 98: 2453–2460. 24337250 10.1007/s00253-013-5401-0

[mbt212453-bib-0026] Ochi, A. , Matsumoto, K.I. , Ooba, T. , Sakai, K. , Tsuge, T. , and Taguchi, S. (2013) Engineering of class I lactate‐polymerizing polyhydroxyalkanoate synthases from *Ralstonia eutropha* that synthesize lactate‐based polyester with a block nature. Appl Microbiol Biotechnol 97: 3441–3447. 22801709 10.1007/s00253-012-4231-9

[mbt212453-bib-0027] Ouyang, S.‐P. , Luo, R.C. , Chen, S.‐S. , Liu, Q. , Chung, A. , Wu, Q. , and Chen, G.‐Q. (2007) Production of polyhydroxyalkanoates with high 3‐hydroxydodecanoate monomer content by fadB and fadA knockout mutant of *Pseudomonas putida* KT2442. Biomacromolecules 8: 2504–2511. 17661516 10.1021/bm0702307

[mbt212453-bib-0028] Park, S.J. , Lee, S.Y. , Kim, T.W. , Jung, Y.K. , and Yang, T.H. (2012a) Biosynthesis of lactate‐containing polyesters by metabolically engineered bacteria. Biotechnol J 7: 199–212. 22057878 10.1002/biot.201100070

[mbt212453-bib-0029] Park, S.J. , Lee, T.W. , Lim, S.‐C. , Kim, T.W. , Lee, H. , Kim, M.K. , *et al* (2012b) Biosynthesis of polyhydroxyalkanoates containing 2‐hydroxybutyrate from unrelated carbon source by metabolically engineered *Escherichia coli* . Appl Microbiol Biotechnol 93: 273–283. 21842437 10.1007/s00253-011-3530-x

[mbt212453-bib-0030] Park, S.J. , Jang, Y.‐A. , Lee, H. , Park, A.‐R. , Yang, J.E. , Shin, J. , *et al* (2013) Metabolic engineering of *Ralstonia eutropha* for the biosynthesis of 2‐hydroxyacid‐containing polyhydroxyalkanoates. Metab Eng 20: 20–28. 23973656 10.1016/j.ymben.2013.08.002

[mbt212453-bib-0031] Park, S.J. , Jang, Y.A. , Noh, W. , Oh, Y.H. , Lee, H. , David, Y. , *et al* (2015) Metabolic engineering of *Ralstonia eutropha* for the production of polyhydroxyalkanoates from sucrose. Biotechnol Bioeng 112: 638–643. 25258020 10.1002/bit.25469

[mbt212453-bib-0032] Povolo, S. , Romanelli, M.G. , Basaglia, M. , Ilieva, V.I. , Corti, A. , Morelli, A. , *et al* (2013) Polyhydroxyalkanoate biosynthesis by *Hydrogenophaga pseudoflava* DSM1034 from structurally unrelated carbon sources. New Biotechnol 30: 629–634. 10.1016/j.nbt.2012.11.019 23201074

[mbt212453-bib-0033] Salamanca‐Cardona, L. , Ashe, C.S. , Stipanovic, A.J. , and Nomura, C.T. (2014) Enhanced production of polyhydroxyalkanoates (PHAs) from beechwood xylan by recombinant *Escherichia coli* . Appl Microbiol Biotechnol 98: 831–842. 24287934 10.1007/s00253-013-5398-4

[mbt212453-bib-0034] Shen, X.‐W. , Yang, Y. , Jian, J. , Wu, Q. , and Chen, G.‐Q. (2009) Production and characterization of homopolymer poly (3‐hydroxyvalerate)(PHV) accumulated by wild type and recombinant *Aeromonas hydrophila* strain 4AK4. Bioresour Technol 100: 4296–4299. 19395256 10.1016/j.biortech.2009.03.065

[mbt212453-bib-0035] Shozui, F. , Matsumoto, K.I. , Motohashi, R. , Yamada, M. , and Taguchi, S. (2010a) Establishment of a metabolic pathway to introduce the 3‐hydroxyhexanoate unit into LA‐based polyesters via a reverse reaction of β‐oxidation in *Escherichia coli* LS5218. Polym Degrad Stab 95: 1340–1344.

[mbt212453-bib-0036] Shozui, F. , Matsumoto, K.I. , Nakai, T. , Yamada, M. , and Taguchi, S. (2010b) Biosynthesis of novel terpolymers poly (lactate‐co‐3‐hydroxybutyrate‐co‐3‐hydroxyvalerate) s in lactate‐overproducing mutant *Escherichia coli* JW0885 by feeding propionate as a precursor of 3‐hydroxyvalerate. Appl Microbiol Biotechnol 85: 949–954. 19582448 10.1007/s00253-009-2100-y

[mbt212453-bib-0037] Shozui, F. , Matsumoto, K.I. , Motohashi, R. , Sun, J. , Satoh, T. , Kakuchi, T. , and Taguchi, S. (2011) Biosynthesis of a lactate (LA)‐based polyester with a 96 mol% LA fraction and its application to stereocomplex formation. Polym Degrad Stab 96: 499–504.

[mbt212453-bib-0038] Silva‐Rocha, R. , Martínez‐García, E. , Calles, B. , Chavarría, M. , Arce‐Rodríguez, A. , de las Heras, A. , *et al* (2013) The Standard European Vector Architecture (SEVA): a coherent platform for the analysis and deployment of complex prokaryotic phenotypes. Nucleic Acids Res 41: D666–D675. 23180763 10.1093/nar/gks1119 PMC3531073

[mbt212453-bib-0039] Simon, R. , Priefer, U. , and Pühler, A. (1983) A broad host range mobilization system for in vivo genetic engineering: transposon mutagenesis in gram negative bacteria. Nat Biotechnol 1: 784–791.

[mbt212453-bib-0040] Song, Y. , Matsumoto, K.I. , Yamada, M. , Gohda, A. , Brigham, C.J. , Sinskey, A.J. , and Taguchi, S. (2012) Engineered *Corynebacterium glutamicum* as an endotoxin‐free platform strain for lactate‐based polyester production. Appl Microbiol Biotechnol 93: 1917–1925. 22127753 10.1007/s00253-011-3718-0

[mbt212453-bib-0041] Spiekermann, P. , Rehm, B.H. , Kalscheuer, R. , Baumeister, D. , and Steinbüchel, A. (1999) A sensitive, viable‐colony staining method using Nile red for direct screening of bacteria that accumulate polyhydroxyalkanoic acids and other lipid storage compounds. Arch Microbiol 171: 73–80. 9914303 10.1007/s002030050681

[mbt212453-bib-0042] Sudesh, K. , and Iwata, T. (2008) Sustainability of biobased and biodegradable plastics. CLEAN Soil Air Water 36: 433–442.

[mbt212453-bib-0043] Taguchi, S. , Yamada, M. , Matsumoto, K.I. , Tajima, K. , Satoh, Y. , Munekata, M. , *et al* (2008) A microbial factory for lactate‐based polyesters using a lactate‐polymerizing enzyme. Proc Natl Acad Sci USA 105: 17323–17327. 18978031 10.1073/pnas.0805653105 PMC2582312

[mbt212453-bib-0044] Tran, T.T. , and Charles, T.C. (2015) Genome‐engineered *Sinorhizobium meliloti* for the production of poly (lactic‐co‐3‐hydroxybutyric) acid copolymer. Can J Microbiol 62: 1–9. 26639519 10.1139/cjm-2015-0255

[mbt212453-bib-0045] Tripathi, L. , Wu, L.‐P. , Chen, J. , and Chen, G.‐Q. (2012) Synthesis of Diblock copolymer poly‐3‐hydroxybutyrate‐block‐poly‐3‐hydroxyhexanoate [PHB‐b‐PHHx] by a beta‐oxidation weakened *Pseudomonas putida* KT2442. Microb Cell Fact 11: 44. 22480145 10.1186/1475-2859-11-44 PMC3442986

[mbt212453-bib-0046] Yamada, M. , Matsumoto, K.I. , Shimizu, K. , Uramoto, S. , Nakai, T. , Shozui, F. , and Taguchi, S. (2010) Adjustable mutations in lactate (LA)‐polymerizing enzyme for the microbial production of LA‐based polyesters with tailor‐made monomer composition. Biomacromolecules 11: 815–819. 20166718 10.1021/bm901437z

[mbt212453-bib-0047] Yamada, M. , Matsumoto, K.I. , Uramoto, S. , Motohashi, R. , Abe, H. , and Taguchi, S. (2011) Lactate fraction dependent mechanical properties of semitransparent poly (lactate‐co‐3‐hydroxybutyrate) s produced by control of lactyl‐CoA monomer fluxes in recombinant *Escherichia coli* . J Biotechnol 154: 255–260. 21640144 10.1016/j.jbiotec.2011.05.011

[mbt212453-bib-0048] Yang, T.H. , Jung, Y.K. , Kang, H.O. , Kim, T.W. , Park, S.J. , and Lee, S.Y. (2011) Tailor‐made type II Pseudomonas PHA synthases and their use for the biosynthesis of polylactic acid and its copolymer in recombinant *Escherichia coli* . Appl Microbiol Biotechnol 90: 603–614. 21221571 10.1007/s00253-010-3077-2

[mbt212453-bib-0049] Zaman, H.U. , Song, J.C. , Park, L.‐S. , Kang, I.‐K. , Park, S.‐Y. , Kwak, G. , *et al* (2011) Poly (lactic acid) blends with desired end‐use properties by addition of thermoplastic polyester elastomer and MDI. Polym Bull 67: 187–198.

[mbt212453-bib-0050] Zhou, L. , Zuo, Z.‐R. , Chen, X.‐Z. , Niu, D.‐D. , Tian, K.‐M. , Prior, B.A. , *et al* (2011a) Evaluation of genetic manipulation strategies on D‐lactate production by *Escherichia coli* . Curr Microbiol 62: 981–989. 21086129 10.1007/s00284-010-9817-9

[mbt212453-bib-0051] Zhou, Q. , Shi, Z.Y. , Meng, D.C. , Wu, Q. , Chen, J.C. , and Chen, G.Q. (2011b) Production of 3‐hydroxypropionate homopolymer and poly(3‐hydroxypropionate‐co‐4‐hydroxybutyrate) copolymer by recombinant *Escherichia coli* . Metab Eng 13: 777–785. 22024131 10.1016/j.ymben.2011.10.002

[mbt212453-bib-0052] Zhu, J. , and Shimizu, K. (2004) The effect of pfl gene knockout on the metabolism for optically pure D‐lactate production by *Escherichia coli* . Appl Microbiol Biotechnol 64: 367–375. 14673546 10.1007/s00253-003-1499-9

