# Supplementary material for: Microbial synthesis of a novel terpolyester P(LA‐co‐3HB‐co‐3HP) from low‐cost substrates
Source: Microb Biotechnol. 2016 Nov 17;10(2):371–80. doi: 10.1111/1751-7915.12453 (PMC5328817; doi:10.1111/1751-7915.12453)
Supplement: Supplementary file 1 — Table S1. Site specific mutations of two PHA synthases phaC Ps variants. Table S2. Comparison of various LA polymerizing enzymes. Table S3. P3HP synthetic ability of E. coli harboring p3HP1p and p3HP2p plasmids, respectively. Table S4. Effects of expressing gene ldhA on LA synthesis. Fig. S1.1. Comparison of plasmids structure between pLA and pLA'. Fig. S1.2. Relationship between pct transcriptional level and LA ratio in the terpolymer. Fig. S2. Formations of extracellular formate and lactate by E. coli S17‐1 (A) and the ΔpflA mutant (B) under aerobic conditions. [file MBT2-10-371-s001.docx]

**Supplementary info:**

**Original paper**

**Title:**

**Microbial Synthesis of A Novel Terpolymer P(LA-*co*-3HB-*co*-3HP) from Unrelated Carbon Sources**

**Authors:**

Yilin Ren^a^, Dechuan Meng^a^, Linping WU^b^, Jinchun Chen^a^, Qiong WU^a^, Guo-Qiang Chen^a,c,d^

**Addresses:**

^a^ Center for Synthetic and Systems Biology, School of Life Science, Tsinghua-Peking Center for Life Sciences, Tsinghua University, Beijing 100084, China

^b^ Department of Pharmacy, Faculty of Health and Medical Sciences, University of Copenhagen, Copenhagen 2100, Denmark

^c^ Center for Nano and Micro Mechanics, Tsinghua University, Beijing 100084, China

^d^ MOE Key Lab of Industrial Biocatalysis, Dept Chemical Engineering, Tsinghua University, Beijing 100084, China

**Supplementary Table I.** Site specific mutations of two PHA synthases *phaC_Ps_* variants.


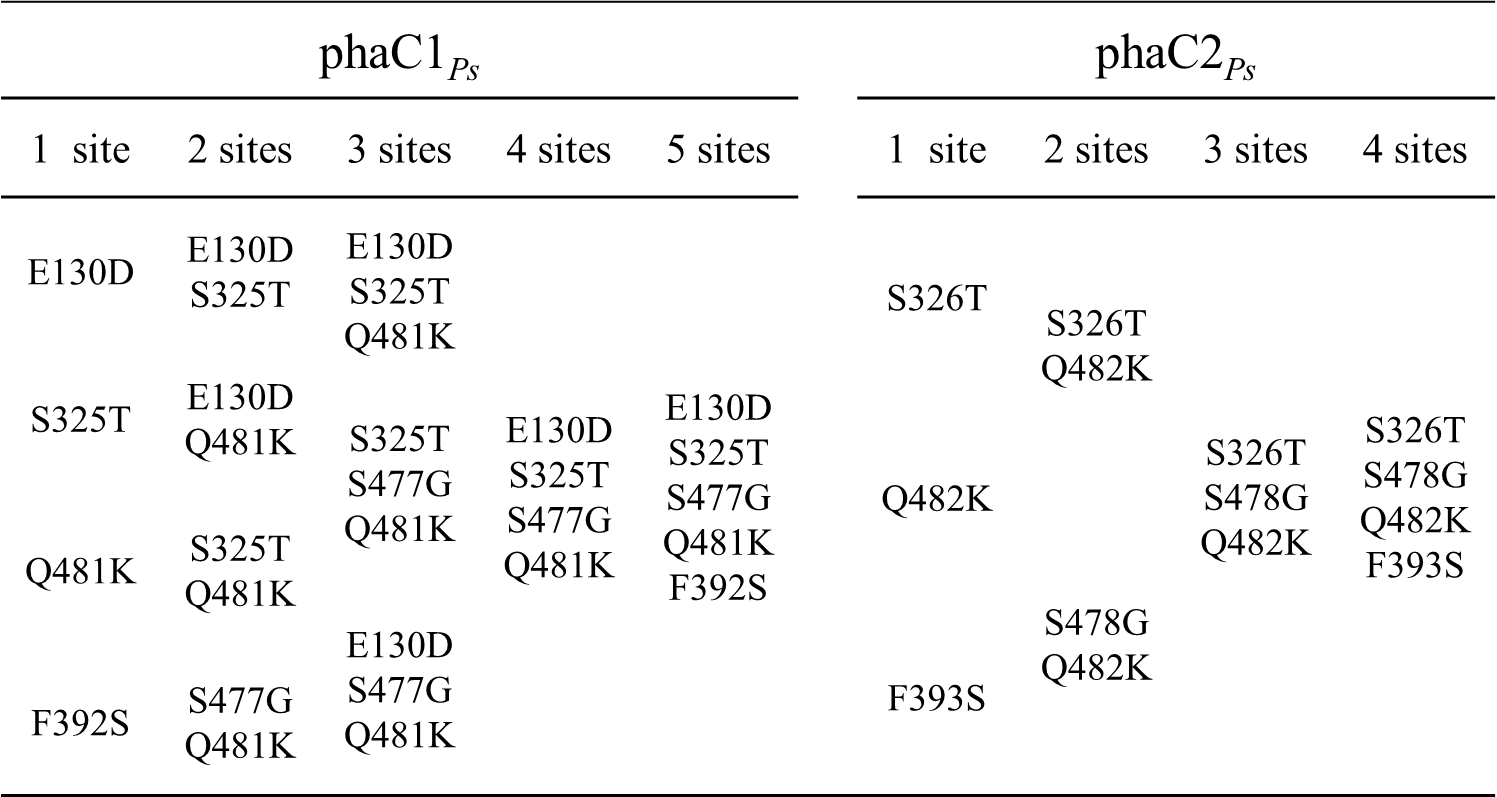


The mutation sites were selected based on alignment with *phaC1_Ps6-19_* of *Pseudomonas sp.6-10* and *phaC1_Ps61-3_* of *Pseudomonas sp.61-3*. Referring to the previous studies, some specific mutations beneficial to broaden the substrate affinities on these sites, were conducted. E130D indicates that the glutamic acid at 130 Amino acid site of phaC1*_Ps_* was replaced by Aspartic acid, etc. Abbreviations: E, glutamic acid; D, aspartic acid; S, serine; F, phenylalanine; Q, glutamine; T, Threonine; K, Lysine; G, Glycine.

**Supplementary Table II.** Comparison of various LA polymerizing enzymes.

| *E. coli* | *phaC* variants | CDW (g/L) | | PHA (wt%) | | LA (mol%) | |  |
| --- | --- | --- | --- | --- | --- | --- | --- | --- |
| S-BLPCAB | *phaC1_Ps_* (Q481K S325T E130D S477G) | | 3.13±0.31 | | 36.94±0.38 | | 5.01±1.24 | |
| S-BLPCAB61-3 | *phaC1_Ps61_*_-3_ (S325T Q481K) | | 3.37±0.92 | | 2.72±1.55 | | - | |
| S-BLPCAB6-19 | *phaC1_Ps6-19_* (E130D S325T S477G Q481K) | | 3.52±0.28 | | 46.64±1.48 | | 1.39±1.07 | |
| S-BLPCABRe | *phaC_Re_* (A510S) | | 5.47±0.32 | | 56.13±1.23 | | - | |

Recombinant strains were cultivated for 48 h. Data shown are the averages and standard deviations of three parallel experiments. *phaC1_Ps61-3_* (S325T Q481K), *phaC1_Ps6-19_* (E130D S325T S477G Q481K) and *phaC_Re_* (A510S) were LA polymerizing enzymes reported in previous studies (Taguchi, *et al*., 2008; Yang, *et al*., 2011; Ochi, *et al*., 2013). Abbreviations: *phaC1_Ps,_* *Pseudomonas stutzeri* *phaC1*; *phaC1_Ps61-3_*, *Pseudomonas sp.61-3 phaC1*; *phaC1_Ps6-19_*, *Pseudomonas sp.6-19 phaC1*; *phaC_Re_*_,_ *Ralstonia eutropha phaC*.

**Supplementary Table III.** P3HP synthetic ability of E. coli harboring p3HP1p and p3HP2p plasmids, respectively.

| Plasmids | CDW (g/L) | PHA (wt%) | 3HB (mol%) | 3HP (mol%) |
| --- | --- | --- | --- | --- |
| pBHR68 | 9.2±0.55 | 64.22±0.53 | 100 | 0 |
| p3HP1p | 3.16±0.08 | 0 | - | - |
| pBHR68+p3HP1p | 4.22±0.38 | 19.23±0.59 | 74.45±3.47 | 25.55±3.47 |
| pBHR68+p3HP2p | 7.33±0.71 | 52.74±15.93 | 59.39±1.12 | 40.61±1.12 |

The plasmid listed in the table were transformed into *E. coli* S17-1. The recombinants were cultivated in LB medium supplemented with 20 g/L glucose and 10 g/L glycerol for 48 h in shake flasks. In p3HP1p plasmid, the genes *dhaT*, *aldD, pcs* and *dhaB* were ligated into the pSEVA351 backbone in the sequence downstream a constitutive promoter. Based on p3HP1p, one more P*_re_* promoter were inserted upstream gene *dhaB* that was a limiting step of glycerol utilization, generating plasmaid p3HP2p. The data are the averages of three parallel experiments. Abbreviations: CDW, cell dry weight; 3HP, 3-hydroxypropionate; 3HB, 3-hydroxybutyrate. There was no PHA accumulation in strain harboring p3HP plasmid alone for p3HP plasmid does not contain a PHA synthase. Obviously, strain harboring p3HP2p plasmid showed an enhanced P3HP synthetic capacity towards p3HP1p plasmid for increasing *dhaB* expression level.

**Supplementary Table IV.** Effects of expressing gene *ldhA* on LA synthesis.

| *E. coli* | Plasmids | LA conc. at 24 h (g/L) | LA conc.at 48 h (g/L) |
| --- | --- | --- | --- |
| S-NC | pBluescript SK^-^ | 3.13±0.31 | 3.14±0.11 |
| S-BL | pBL | 3.44±0.15 | 3.25±0.62 |
| S-LA | pLA | 2.80±0.03 | 3.51±0.19 |
| S-LA’ | pLA’ | 3.68±0.37 | 3.22±0.27 |

Strains were cultured in LB medium supplemented with 20 g/L glucose for 48 h. LA concentration was assayed via HPLC with the samples at 24 h and 48 h. Data shown are the averages and standard deviations of three parallel experiments. pBluescript SK^-^ is the commonly used commercial plasmid. pBL plasmid was based on pBluescript SK^-^ with an insertion of P*_re_* promoter upstream *ldhA* gene.

**
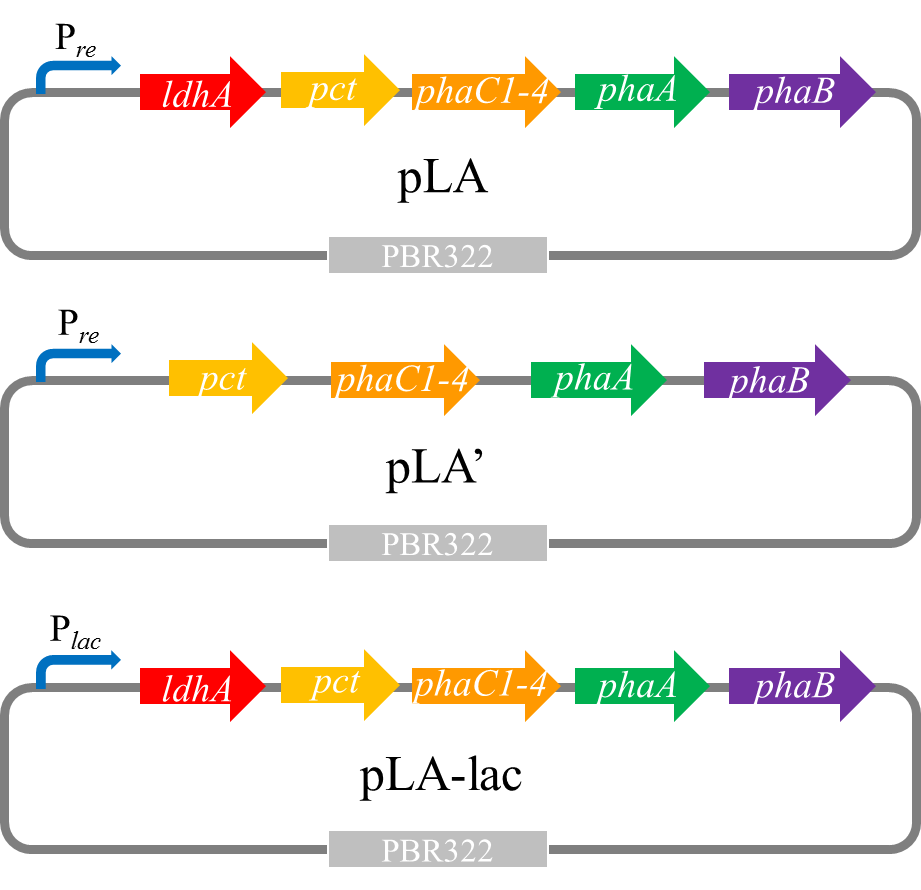
Supplementary Figure 1.1**. Comparison of plasmids structure between pLA and pLA’.

The plasmid pLA’ was derived from pLA by removing gene *ldhA*. The plasmid pLA-lac was also derived from pLA by replacing P*_re_* promoter with P*_lac_* promoter. Abbreviations: P*_re_*, promoter of *R. eutropha* phaCAB operon; *ldhA*, lactate dehydrogenase; *pct*, propionyl-CoA transferase; *phaC1-4*, engineered PHA synthetase PhaC1*_Ps_*(Q481K S325T E130D S477G); *phaA*, β-ketothiolase; *phaB*, NADPH-dependent acetoacetyl-CoA reductase; PBR322, the replicon of plasmid pBluescript SK^-^.


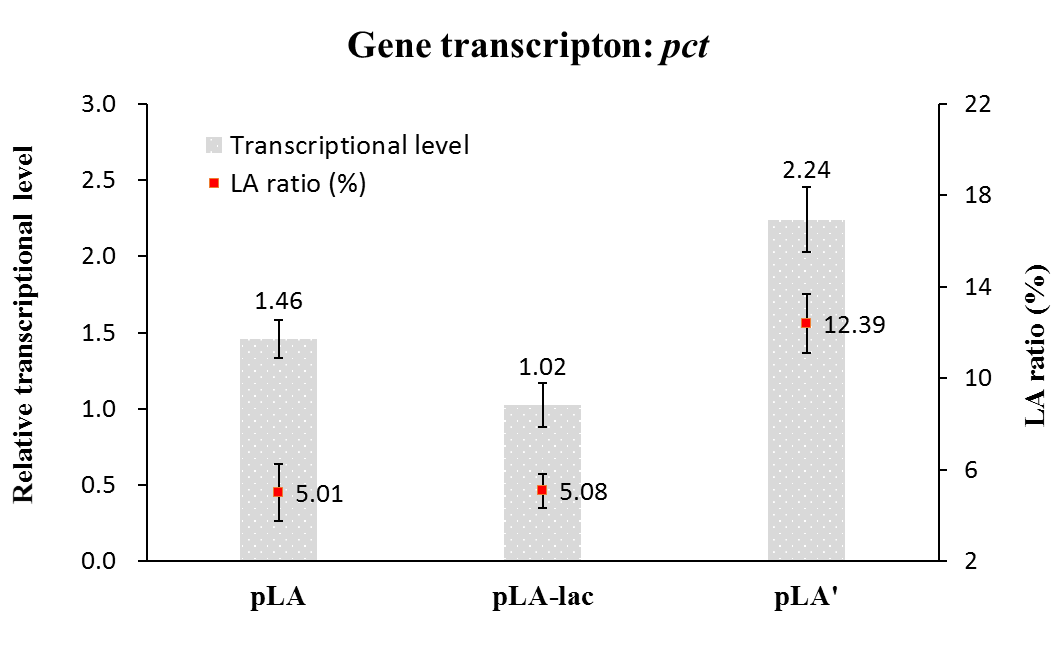


**Supplementary Figure 1.2.** Relationship between *pct* transcriptional level and LA ratio in the terpolymer.

Recombinant *E. coli* S17-1 harboring pLA, pLA’ or pLA-lac plasmid was cultured in LB medium supplemented with 20 g/L glucose. The LA ratio was determined by GC and transcriptional level of *pct* was measured by RT-PCR.


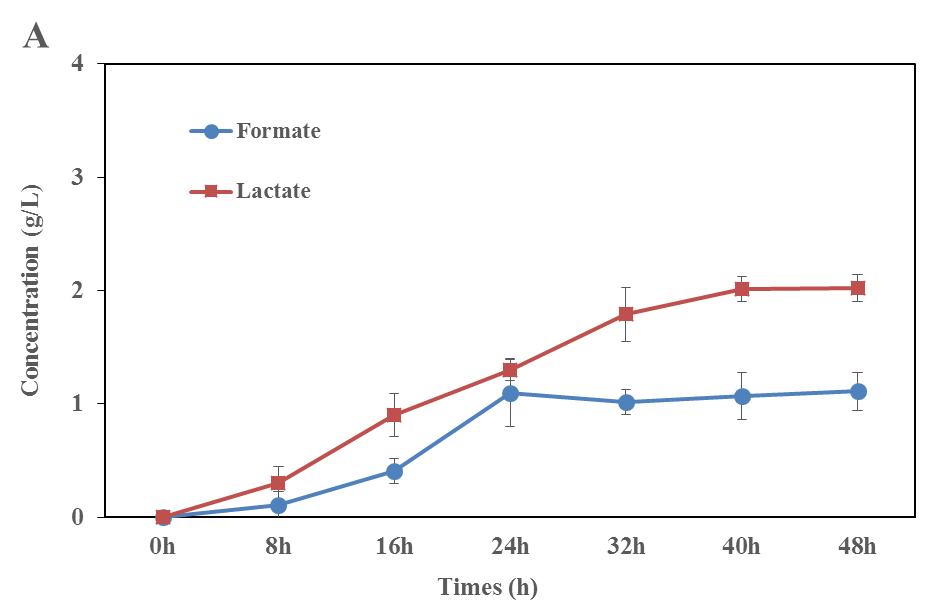

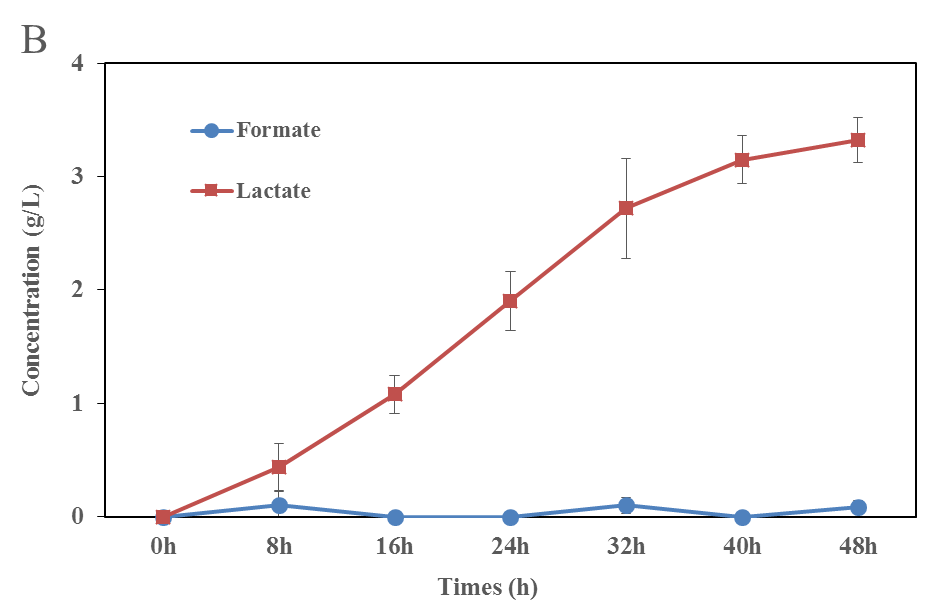
**Supplementary Figure 2.** Formations of extracellular formate and lactate by *E. coli* S17-1 (A) and the Δ*pflA* mutant (B) under aerobic conditions.

Strains A, *E. coli* S17-1, and B *E. coli* S17-1 Δ*pflA,* were cultured in LB medium with 200 rpm for 48 h. Blue solid dot, formate; red solid square, lactate. Error bars represent the standard deviation of experiments conducted in triplicates.

**Reference**

Ochi, A., Matsumoto, K.i., Ooba, T., Sakai, K., Tsuge, T., and Taguchi, S. (2013) Engineering of class I lactate-polymerizing polyhydroxyalkanoate synthases from Ralstonia eutropha that synthesize lactate-based polyester with a block nature, *Appl Microbiol Biotechnol* **97**: 3441-3447.

Taguchi, S., Yamada, M., Matsumoto, K.i., Tajima, K., Satoh, Y., Munekata, M., et al. (2008) A microbial factory for lactate-based polyesters using a lactate-polymerizing enzyme, *Proc Natl Acad Sci USA* **105**: 17323-17327.

Yang, T.H., Jung, Y.K., Kang, H.O., Kim, T.W., Park, S.J., and Lee, S.Y. (2011) Tailor-made type II Pseudomonas PHA synthases and their use for the biosynthesis of polylactic acid and its copolymer in recombinant Escherichia coli, *Appl Microbiol Biotechnol* **90**: 603-614.
